# Combined handgrip strength and incident chronic digestive diseases: a prospective parallel analysis examining broad and specific endpoints in Chinese and European older adults

**DOI:** 10.1080/16549716.2026.2686499

**Published:** 2026-06-15

**Authors:** Jian Liu, Minghao Ji, Xinlong Pang, Xiangyan Liu, Wentao Ji, Zhongwei Xin, Mo Shi

**Affiliations:** a Department of Thoracic Surgery, Shandong Provincial Hospital, Shandong First Medical University, Jinan, Shandong, China; b Shandong Academy of Medical Sciences, Shandong First Medical University, Jinan, Shandong, China

**Keywords:** Digestive system diseases, hand strength, sarcopenia, aged, peptic ulcer

## Abstract

**Background:**

Handgrip strength is a simple marker of muscle function, but its prospective association with digestive morbidity remains unclear.

**Objective:**

To examine the association between combined handgrip strength (CHS) and incident self-reported digestive outcomes in two ageing cohorts with different endpoint definitions.

**Methods:**

This prospective parallel analysis included 7,750 participants from CHARLS (2011–2018) and 6,728 from SHARE (2011–2017), after excluding individuals with baseline digestive diseases. CHS was the sum of the maximum grip strength values from both hands. Outcomes were incident chronic digestive system diseases in the China Health and Retirement Longitudinal Study (CHARLS) and incident peptic ulcer disease in the Survey of Health, Ageing and Retirement in Europe (SHARE), based on self-reported physician diagnoses. Cox proportional hazards models were used, with sensitivity analyses using shorter follow-up windows and maximum single-hand grip strength.

**Results:**

In CHARLS, higher CHS was associated with lower risk of incident chronic digestive system diseases in the fully adjusted model (HR per 1 kg increase, 0.990; 95% CI, 0.984–0.997), and the high-CHS group had lower risk than the low-CHS group (HR, 0.895; 95% CI, 0.803–0.998). In SHARE, the overall association with peptic ulcer disease was weaker and non-significant (HR, 0.996; 95% CI, 0.991–1.001), whereas an inverse association was observed among women (HR, 0.989; 95% CI, 0.979–0.999). Sensitivity analyses were generally directionally consistent.

**Conclusions:**

Lower CHS was associated with subsequent self-reported digestive outcomes, more consistently in CHARLS than in SHARE. Outcome heterogeneity, self-reported diagnoses, modest discriminatory performance, and unmeasured confounding warrant cautious interpretation.

## Paper Context

**Main findings:** Lower combined handgrip strength was associated with subsequent self-reported digestive outcomes in two ageing cohorts, with stronger evidence for broad chronic digestive system diseases in the China Health and Retirement Longitudinal Study than for peptic ulcer disease in the Survey of Health, Ageing and Retirement in Europe.

**Added knowledge:** This study adds population-level prospective evidence that the association between muscle strength and digestive morbidity varies by outcome definition and population context.

**Global health impact for policy and action:** Handgrip strength may be further evaluated as a low-cost marker of physiological vulnerability in ageing populations, but integration into risk-assessment frameworks requires validation using clinically confirmed outcomes and detailed medication, infection, and nutritional data.

## Background

Chronic digestive diseases constitute a substantial and growing public health burden worldwide. This broad category encompasses a wide spectrum of conditions, ranging from functional gastrointestinal disorders, such as irritable bowel syndrome, to organic diseases, including chronic gastritis, inflammatory bowel disease, peptic ulcer disease, and liver cirrhosis [1–3]. Beyond their direct clinical consequences, chronic digestive diseases are characterised by long-term management needs, frequent recurrence, and substantial reductions in quality of life, together creating sustained demand on health-care resources and imposing socioeconomic burden across both high- and middle-income countries [4–6]. In China, chronic digestive diseases are estimated to affect more than one-third of the adult population, and their burden is expected to increase further in the context of population ageing [7,8]. This growing burden highlights the need to identify simple and scalable markers that may help characterise vulnerability to digestive morbidity, particularly among middle-aged and older adults. Within this broad spectrum, peptic ulcer disease is a clinically recognisable organic digestive disorder that warrants specific epidemiological attention. It shares several features with chronic digestive diseases more broadly, including recurrence, long-term clinical impact, and reduced health-related quality of life, while also representing a more specific upper gastrointestinal endpoint available in large population-based cohorts. Global Burden of Disease estimates indicate that peptic ulcer disease remains an important contributor to digestive disease burden, particularly in ageing populations [4–6,9,10]. Accordingly, examining both broad chronic digestive outcomes and more specific organic digestive endpoints may provide complementary, although not directly interchangeable, perspectives on the relationship between systemic health status and digestive morbidity. Handgrip strength is a simple, non-invasive, and inexpensive measure that is widely recognised as a reliable biomarker of overall muscle strength and physiological reserve [11–14]. Substantial prospective evidence has linked low handgrip strength to multiple adverse health outcomes, including all-cause mortality, cardiovascular disease, respiratory illness, several cancers, and type 2 diabetes [11,14–18]. Reflecting this clinical relevance, handgrip strength is embedded in the operational definition of sarcopenia by authoritative bodies such as the European Working Group on Sarcopenia in Older People, and is increasingly regarded as a practical indicator of general health status rather than of muscle function alone [13]. Despite well-documented links between muscle strength and a range of multi-system chronic conditions, evidence connecting handgrip strength with incident chronic digestive diseases remains limited and heterogeneous. Although a recent prospective cohort study linked skeletal muscle strength and mass with subsequent gastrointestinal disease risk, much of the related literature has focused on general muscle measures or prognostic assessment within specific patient populations [19,20]. In particular, the prospective association between combined handgrip strength (CHS) – defined as the sum of the maximum values from both hands and used here as a bilateral measure of muscle function – and incident digestive outcomes has not been systematically examined in large general-population cohorts [12,21]. To address this gap, we used data from two large, independent prospective cohorts – the China Health and Retirement Longitudinal Study (CHARLS) and the Survey of Health, Ageing and Retirement in Europe (SHARE) – to conduct a parallel analysis of the association between baseline CHS and incident self-reported digestive outcomes [22,23]. In the Chinese cohort, we examined incident chronic digestive system diseases, a broad self-reported endpoint capturing heterogeneous functional and organic digestive conditions. In the European cohort, we examined incident peptic ulcer disease, a more specific self-reported organic endpoint available in that cohort. Because these outcomes differ in scope and definition, the European analysis was not intended as a direct replication of the Chinese analysis. Instead, the two cohorts were used to provide complementary evidence on whether lower muscle strength is associated with subsequent digestive morbidity across different ageing populations and outcome definitions.

## Methods

### Study design and population

This study used a parallel cohort design and was based on a secondary analysis of data from two large, multicentre prospective cohorts: CHARLS and SHARE. These high-quality public databases were selected because they provide standardised, nationally representative data for investigating the association between muscle strength and distinct digestive health outcomes in two populations with different socioeconomic and ethnic backgrounds [22,23]. For the Chinese population, we used data from the 2011 national baseline survey of CHARLS, which was longitudinally integrated with follow-up waves in 2013, 2015, and 2018. For the European population, data from SHARE Waves 4 (2011) to 7 (2017) were selected to ensure a comparable time frame.

**Figure 1.**
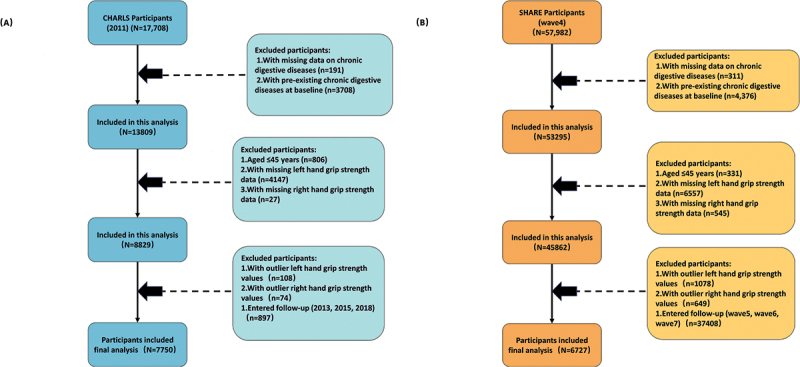
Flowchart of participant selection from (A) CHARLS and (B) SHARE. Covariate missingness within the final samples was handled by multiple imputation, as described in the methods.

Participants aged 45 years or older were included from both databases. Individuals were excluded if they reported a history of chronic digestive system diseases (in CHARLS) or peptic ulcer disease (in SHARE) at baseline, had missing data on handgrip strength, had physiologically implausible grip strength values (<5 kg or >90 kg, defined a priori as outliers), or had incomplete follow-up data. After this screening process, 7,750 participants from CHARLS and 6,728 participants from SHARE were included in the final analytical samples (Figure 1).

Within these final samples, residual missingness in covariates was addressed using multiple imputation by chained equations under a missing-at-random assumption. Imputation was performed separately for each cohort, with all baseline covariates and the outcome event indicator included as predictors; five imputed datasets were generated, and estimates from each were combined using Rubin’s rules. Sensitivity exposure variables (CHS and maximum single-hand grip strength [MSHS)]) were not imputed and were used as observed.

Both CHARLS and SHARE were approved by their respective institutional ethics committees, and all participants provided written informed consent prior to enrolment; full ethics approval details are provided in the Ethics and consent statement.

### CHS assessment

The primary exposure of this study was CHS, defined as the sum of the maximum handgrip strength values measured from the left and right hands at baseline, expressed in kilograms. To enable a direct comparison with the conventional unilateral approach, we additionally evaluated MSHS, defined as the higher of the left or right hand maximum values, as a sensitivity exposure restricted to the overall population of each cohort. In CHARLS, handgrip strength was measured using a Yuejian™ WL-1000 dynamometer; in SHARE, measurements were obtained using a Smedley portable dynamometer. In both cohorts, participants performed two maximal-effort attempts with each hand, and the higher value per hand was retained for analysis; the maximum left and right values were then used to derive CHS (the sum of the two hands) and MSHS (the higher of the two hands), both expressed in kilograms. No body-weight correction was applied to the grip strength values used in either exposure variable; body mass index was instead included as a covariate in the fully adjusted Cox regression models to account for body size differences.

### Ascertainment of incident outcomes

We adopted cohort-specific outcome definitions reflecting the digestive disease information available in each dataset. In CHARLS, the primary outcome was incident chronic digestive system diseases, ascertained by the question, ‘Has a doctor ever told you that you were diagnosed with chronic digestive system diseases?’ In SHARE, the primary outcome was incident peptic ulcer disease (PUD), a more specific organic digestive endpoint available in that cohort, captured by the question, ‘Has a doctor ever told you that you have a stomach or duodenal ulcer, also called peptic ulcer?’ In both cohorts, outcome ascertainment relied on participants’ self-reported physician diagnoses recorded at each follow-up wave. An incident case was defined as a participant who reported no diagnosis at baseline but reported a new diagnosis at any subsequent wave [22,23]. Because incident diagnoses were ascertained at discrete survey waves rather than on the actual date of disease onset, time-to-event was approximated as the interval between the baseline year and the year of the wave at which the new diagnosis was first reported. Participants who remained free of the outcome were right-censored at the year of their last completed survey. This approach approximates interval-censored data, and the robustness of findings against this approximation was further evaluated through two pre-specified sensitivity analyses restricted to shorter follow-up windows. Because the broad CHARLS endpoint and the specific SHARE endpoint differ in scope and clinical definition, the two analyses were not designed to provide a direct replication of one another but rather to provide complementary evidence on the association between handgrip strength and chronic digestive disease morbidity at two levels of outcome specificity in two distinct ageing populations.

### Covariates

Baseline covariates were collected from both cohorts to address potential confounding in the association between handgrip strength and incident outcomes and were organised into four domains: sociodemographic characteristics (age, sex, marital status, and residence, with educational attainment additionally available in SHARE); lifestyle factors (smoking status and alcohol consumption); anthropometry (body mass index, used to account for differences in body size between participants); and self-reported clinical comorbidities. The clinical comorbidity panel was harmonised between cohorts to the extent permitted by data availability. In CHARLS, the panel comprised hypertension, dyslipidemia, diabetes, heart disease, stroke, kidney disease, chronic lung disease, psychiatric disease, and arthritis. In SHARE, it comprised hypertension, diabetes, heart disease, stroke, kidney disease, chronic lung disease, psychiatric disease, and arthritis. The two cohorts thus shared an aligned core set of comorbidities, differing only in the cohort-specific availability of dyslipidemia (CHARLS only) and educational attainment (SHARE only). All covariates were measured at the baseline wave, prior to the ascertainment of incident outcomes during follow-up.

### Statistical analysis

All statistical analyses were performed using R software (version 4.3.1), with a two-sided *p* < 0.05 considered statistically significant. Baseline characteristics were summarised as mean ± standard deviation for continuous variables and frequency (percentage) for categorical variables, and compared between sexes using independent t-tests and chi-squared tests as appropriate. The dose–response relationship between CHS and incident outcomes was examined using restricted cubic splines. Sex-specific cut-off values of CHS for incident disease were derived from receiver operating characteristic (ROC) curve analysis. Because ROC-based thresholds do not incorporate follow-up time, they are presented as exploratory reference values rather than diagnostic cut-offs. Participants were dichotomised into high- and low-CHS groups based on these thresholds, and cumulative incidence was compared between groups using Kaplan–Meier curves with log-rank tests.

Multivariable Cox proportional hazards models were constructed to quantify the association between CHS and incident outcomes. Model 1 was the unadjusted crude model. Model 2 was the fully adjusted model, including age, sex, marital status, residence, smoking status, alcohol consumption, body mass index, and the full panel of clinical comorbidities described above (with educational attainment additionally adjusted in the SHARE cohort). For sex-stratified analyses, sex was excluded from the adjustment set. CHS was modelled both as a continuous variable (per 1 kg increase) and as a dichotomous variable based on the sex-specific ROC-derived cut-offs. As an additional sensitivity exposure, MSHS was analysed in the overall population using the same Model 2 specification. Handgrip strength asymmetry was also examined as an additional sensitivity analysis and is reported in the supplementary materials. To assess the robustness of our findings to the use of self-reported outcomes ascertained at discrete follow-up waves, two pre-specified sensitivity analyses were conducted using the same covariate set as Model 2. The first restricted outcome ascertainment to the first follow-up wave (2011–2013), evaluating the short-term association. The second extended the analysis window to the first two follow-up waves (2011–2015), testing stability over the medium term.

The proportional hazards assumption underlying Model 2 was assessed using scaled Schoenfeld residuals via the cox.zph() function in the R ‘survival’ package. No significant violation was observed for the primary exposure, CHS, or in the global model test (CHARLS: *P*_CHS = 0.418, *P*_global = 0.276; SHARE: *P*_CHS = 0.563, *P*_global = 0.341), supporting the use of the Cox proportional hazards specification. Finally, pre-specified subgroup analyses were conducted in both cohorts to explore potential effect modification. The statistical significance of interactions was tested by including a multiplicative term between CHS and the covariate of interest in the fully adjusted model.

## Results

### Baseline characteristics of participants

This parallel analysis was based on 7,750 participants from the CHARLS cohort and 6,728 from the SHARE cohort. The two cohorts exhibited significant differences in demographic characteristics, lifestyle factors, and clinical comorbidities (Table 1 and Table S1). The CHARLS cohort was relatively younger, with a mean age of 57.6 ± 9.0 years, representing a middle-aged and older Chinese population. As shown in Table 1, significant sex-based differences were present within this cohort. Male participants were older and had substantially higher rates of smoking (73.3% vs. 7.0%) and alcohol consumption (66.6% vs. 14.4%) compared with females. Conversely, female participants had a higher mean BMI (25.0 vs. 23.3 kg/m^2^) and a greater prevalence of arthritis (31.9% vs. 20.3%). In contrast, the SHARE cohort was older (mean age 65.9 ± 8.0 years) and had a higher burden of comorbidities. Table S1 shows that the prevalence of hypertension (44.5%), diabetes (11.7%), and heart disease (12.9%) was considerably higher than in the CHARLS cohort. The pattern of sex-based differences was similar to that in CHARLS, with males having higher rates of smoking and alcohol consumption. However, unlike the CHARLS cohort, men in the SHARE cohort had a slightly higher mean BMI than women (27.1 vs. 26.3 kg/m^2^).

**Table 1.** Baseline characteristics of participants in the CHARLS cohort (*n* = 7,750).

Characteristic	Overall (n = 7,750)	Women (n = 4,170)	Men (n = 3,580)	*p* value
Age (years), mean ± SD	57.6 ± 9.0	57.0 ± 9.4	58.2 ± 8.6	<0.001
Married, n (%)	6,606 (85.2)	3,418 (82.0)	3,188 (89.1)	<0.001
Urban Residence, n (%)	2,708 (34.9)	1,484 (35.6)	1,224 (34.2)	0.002
Smoking, n (%)	2,914 (37.6)	290 (7.0)	2,624 (73.3)	<0.001
Drinking, n (%)	2,985 (38.5)	602 (14.4)	2,383 (66.6)	<0.001
BMI (kg/m^2^), mean ± SD	24.2 ± 4.4	25.0 ± 4.2	23.3 ± 4.6	<0.001
Hypertension, n (%)	1,819 (23.5)	1,042 (25.0)	777 (21.7)	0.011
Dyslipidemia, n (%)	645 (8.3)	362 (8.7)	283 (7.9)	0.521
Diabetes, n (%)	393 (5.1)	230 (5.5)	163 (4.6)	0.468
Stroke, n (%)	151 (2.0)	75 (1.8)	76 (2.8)	<0.001
Heart Disease, n (%)	661 (8.5)	399 (9.6)	262 (7.3)	0.094
Kidney Disease, n (%)	306 (4.0)	142 (3.4)	164 (4.6)	0.024
Lung Disease, n (%)	569 (7.3)	250 (6.0)	319 (8.9)	<0.001
Psychiatric Disease, n (%)	72 (0.9)	45 (1.1)	27 (0.8)	0.478
Arthritis, n (%)	2,209 (28.5)	1,338 (31.9)	871 (20.3)	<0.001

Note. Data are presented as mean ± standard deviation (SD) or number (n) with percentage (%). *p* values were calculated using the independent t-test for continuous variables and the chi-squared (χ^2^) test for categorical variables to compare differences between women and men.

These differences in baseline characteristics provide context for interpreting the parallel analyses. The younger CHARLS cohort, with a lower comorbidity burden, allowed evaluation of the association between CHS and the broad outcome of ‘chronic digestive system diseases.’ The older SHARE cohort, with a more complex clinical profile, provided a complementary setting for evaluating the association between CHS and the more specific endpoint of PUD.

### Dose–response relationship

**Figure 2.**
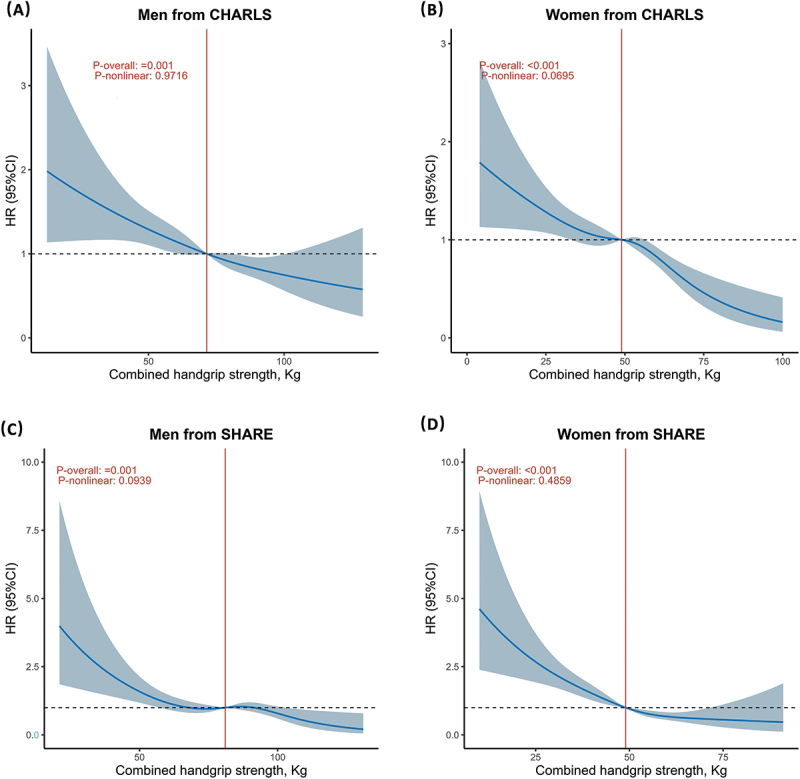
Dose–response relationship between combined handgrip strength and study outcomes, stratified by cohort and sex. (A) Men from the CHARLS cohort with incident chronic digestive system diseases. (B) Women from the CHARLS cohort with incident chronic digestive system diseases. (C) Men from the SHARE cohort with incident peptic ulcer. (D) Women from the SHARE cohort with incident peptic ulcer.

Restricted cubic spline analysis was used to examine the dose–response relationship between CHS and the risk of digestive outcomes. In the CHARLS cohort, CHS showed a significant inverse association with the risk of incident chronic digestive system diseases (Figure 2A,B). This association was observed in both men (*P_*overall = 0.001; *P_*nonlinearity = 0.9716) and women (*P_*overall < 0.001; *P_*nonlinearity = 0.0695). Similarly, in the SHARE cohort, incident PUD risk showed a significant and approximately linear inverse relationship with CHS (Figure 2C,D). This pattern was observed in both men (*P_*overall = 0.001; *P_*nonlinearity = 0.0939) and women (*P_*overall < 0.001; *P_*nonlinearity = 0.4859).

### Risk stratification and cut-off value exploration based on handgrip strength

**Figure 3.**
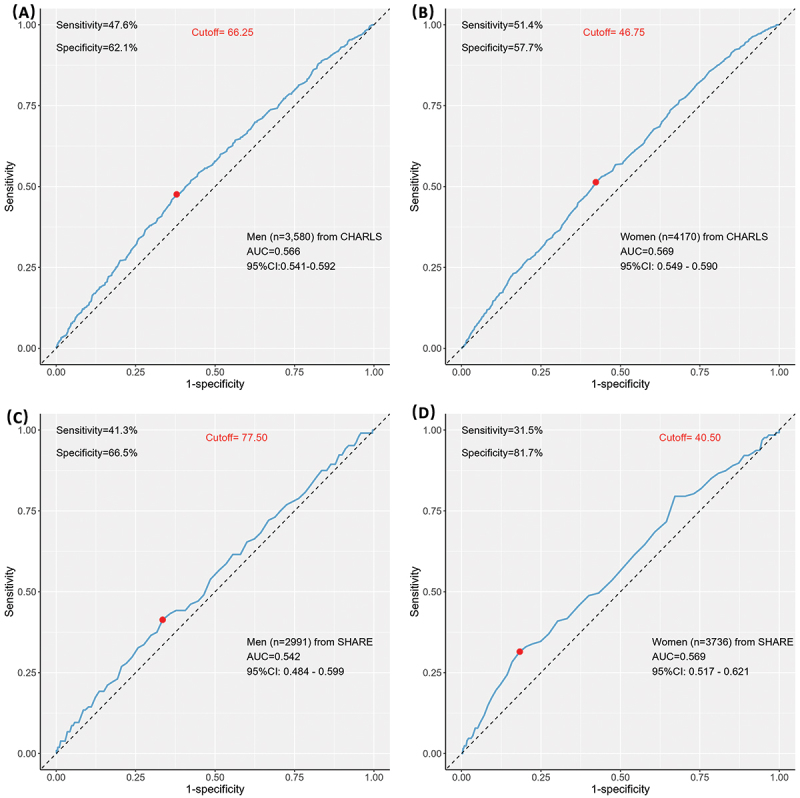
Receiver operating characteristic (ROC) curves for combined handgrip strength in predicting study outcomes, stratified by cohort and sex. (A) Men from the CHARLS cohort with incident chronic digestive system diseases. (B) Women from the CHARLS cohort with incident chronic digestive system diseases. (C) Men from the SHARE cohort with incident peptic ulcer. (D) Women from the SHARE cohort with incident peptic ulcer.

To provide exploratory, data-driven reference values for participant stratification, ROC curve analyses were performed to identify sex-specific CHS cut-off values for the corresponding incident outcome in each cohort. In the CHARLS cohort, the cut-off values for incident chronic digestive system diseases were 66.25 kg for men (Figure 3A) and 46.75 kg for women (Figure 3B). In the SHARE cohort, the corresponding cut-off values for incident PUD were 77.50 kg for men (Figure 3C) and 40.50 kg for women (Figure 3D). These thresholds were used solely to categorise participants into high- and low-CHS groups for within-study analyses and should be interpreted as exploratory, cohort-specific values rather than clinically validated diagnostic cut-offs.

### Cox regression and sensitivity analyses

After confirming that the proportional hazards assumption was satisfied in both cohorts, Cox regression analyses were performed. In the CHARLS cohort, CHS was significantly and inversely associated with the risk of incident chronic digestive system diseases (Table 2). When CHS was modelled as a continuous variable in the fully adjusted Model 2, each 1 kg increment was associated with a significantly lower risk of disease in the overall population (HR, 0.990; 95% CI, 0.984–0.997), as well as in men (HR, 0.991; 95% CI, 0.984–0.998) and women (HR, 0.985; 95% CI, 0.974–0.993). When CHS was analysed categorically, participants in the high-CHS group had an approximately 10.5% lower risk of developing chronic digestive system diseases compared with the low-CHS reference group (HR, 0.895; 95% CI, 0.803–0.998). The two pre-specified sensitivity analyses restricting outcome ascertainment to the first follow-up wave and to the first two follow-up waves yielded estimates directionally consistent with the primary fully adjusted analysis (Table 2). The first-wave overall continuous estimate was attenuated and was not statistically significant (HR, 0.989; 95% CI, 0.975–1.004); however, the inverse association remained statistically significant in women, in the high-CHS group, and in the longer two-wave sensitivity analysis. A parallel sensitivity analysis using MSHS as the exposure yielded directionally similar estimates across the unadjusted, fully adjusted, and follow-up-restricted models (Table 2), suggesting that the findings were not specific to the bilateral grip measure.

**Table 2.** Association of combined handgrip strength and maximal single-hand grip strength with incident chronic digestive system diseases in the CHARLS cohort.

		Main analysis			Sensitivity analyses			
Exposure	Population	n/events	Model 1 HR (95% CI)	Model 2 HR (95% CI)	n/events	First follow-up wave HR (95% CI)	n/events	First two follow-up waves HR (95% CI)
CHS, continuous	Overall	7,750/1,001	0.973 (0.954–0.994)	0.990 (0.984–0.997)	7,750/304	0.989 (0.975–1.004)	7,750/663	0.986 (0.977–0.996)
	Men	3,580/503	0.979 (0.959–0.998)	0.991 (0.984–0.998)	3,580/103	0.995 (0.976–1.015)	3,580/264	0.991 (0.980–1.002)
	Women	4,170/498	0.970 (0.951–0.992)	0.985 (0.974–0.993)	4,170/201	0.982 (0.967–0.998)	4,170/399	0.981 (0.970–0.993)
CHS group	Low	2,397/494	Reference	Reference	2,397/210	Reference	2,397/398	Reference
	High	5,353/507	0.732 (0.566–0.955)	0.895 (0.803–0.998)	5,353/94	0.814 (0.666–0.994)	5,353/271	0.702 (0.584–0.843)
MSHS, continuous	Overall	7,750/1,001	0.966 (0.946–0.988)	0.982 (0.969–0.996)	7,750/304	0.984 (0.963–1.006)	7,750/663	0.980 (0.966–0.994)

Note. Data are presented as hazard ratios (95% confidence intervals). Model 1 was the unadjusted crude model. Model 2 was the fully adjusted model including age, sex, marital status, residence, smoking status, alcohol consumption, body mass index, hypertension, dyslipidemia, diabetes, heart disease, stroke, kidney disease, chronic lung disease, psychiatric disease, and arthritis. In sex-stratified analyses, sex was not included as an adjustment variable. Sensitivity analyses used the same covariates as Model 2 but restricted outcome ascertainment to the first follow-up wave or to the first two follow-up waves. CHS was defined as the sum of the maximum grip strength values from the left and right hands. MSHS was defined as the maximum grip strength value from either hand. The MSHS analysis is presented as a simplified overall-level sensitivity exposure using the same column structure as the CHS analyses. CHS, combined handgrip strength; MSHS, maximal single-hand grip strength; HR, hazard ratio; CI, confidence interval; CHARLS, China Health and Retirement Longitudinal Study.

In the SHARE cohort, the association between CHS and incident PUD varied by sex (Table S2). When CHS was treated as a continuous variable in the fully adjusted Model 2, a statistically significant inverse association was observed among women (HR, 0.989; 95% CI, 0.979–0.999), whereas the associations in the overall population (HR, 0.996; 95% CI, 0.991–1.001) and in men (HR, 0.990; 95% CI, 0.976–1.004) did not reach statistical significance. The categorical analysis showed a similar pattern, with the high-CHS group having a non-significantly lower risk than the low-CHS reference group (HR, 0.785; 95% CI, 0.614–1.005). In the sensitivity analysis restricted to the first follow-up wave, the high-CHS group had a significantly lower risk than the low-CHS group (HR, 0.668; 95% CI, 0.461–0.968). The MSHS sensitivity analysis yielded estimates directionally consistent with the CHS analyses across all model specifications (Table S2). In an additional sensitivity analysis using handgrip strength asymmetry, defined as an asymmetry ratio >1.10, asymmetry was not significantly associated with incident outcomes after full adjustment in either cohort (CHARLS: HR, 1.08; 95% CI, 0.95–1.23; SHARE: HR, 1.07; 95% CI, 0.90–1.27; Table S3). As with CHS, the MSHS association in the overall SHARE population was attenuated after full adjustment but remained statistically significant in the unadjusted model and in the first-wave sensitivity analysis, consistent with the pattern observed for CHS.

### Survival analysis

**Figure 4.**
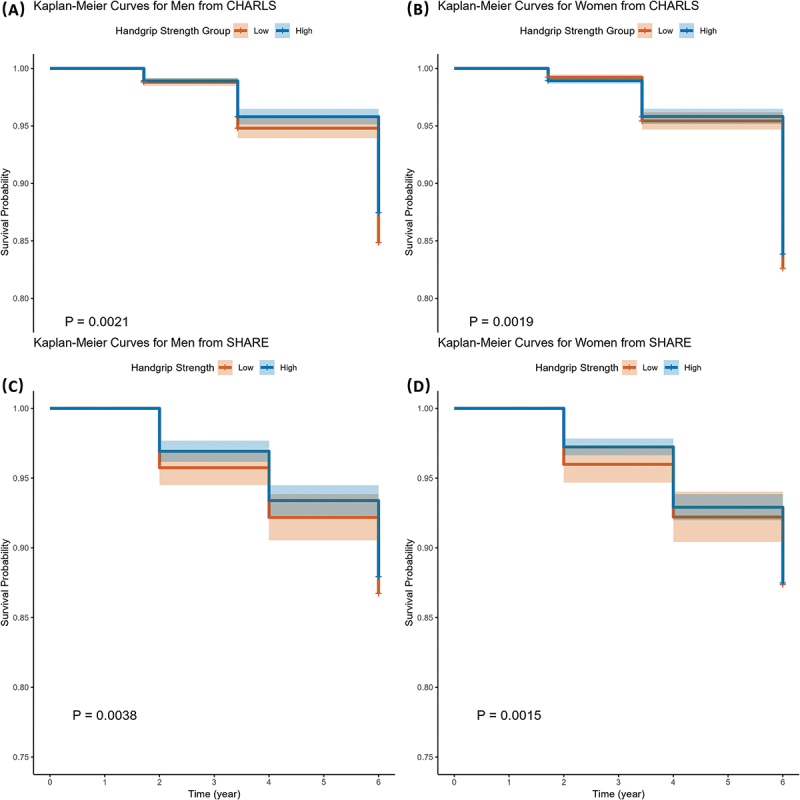
Kaplan–Meier survival curves for study outcomes, stratified by cohort and sex. (A) Men from the CHARLS cohort with incident chronic digestive system diseases. (B) Women from the CHARLS cohort with incident chronic digestive system diseases. (C) Men from the SHARE cohort with incident peptic ulcer. (D) Women from the SHARE cohort with incident peptic ulcer.

To visually assess the cumulative risk of digestive diseases according to handgrip strength level, Kaplan–Meier survival analysis was performed (Figure 4). In the CHARLS cohort, the survival curves (Figure 4A,B) showed that the low-CHS group had a significantly lower probability of remaining free from incident chronic digestive system diseases compared with the high-CHS group. This difference was statistically significant in both men (log-rank *p* = 0.0021) and women (log-rank *p* = 0.0019), with the curves beginning to diverge early in the follow-up period, indicating a sustained risk difference. A similar pattern was observed in the SHARE cohort’s analysis of incident PUD (Figure 4C,D), where men (log-rank *p* = 0.0038) and women (log-rank *p* = 0.0015) in the low-CHS group also exhibited a significantly higher cumulative incidence of the disease.

### Subgroup and interaction analyses

**Figure 5.**
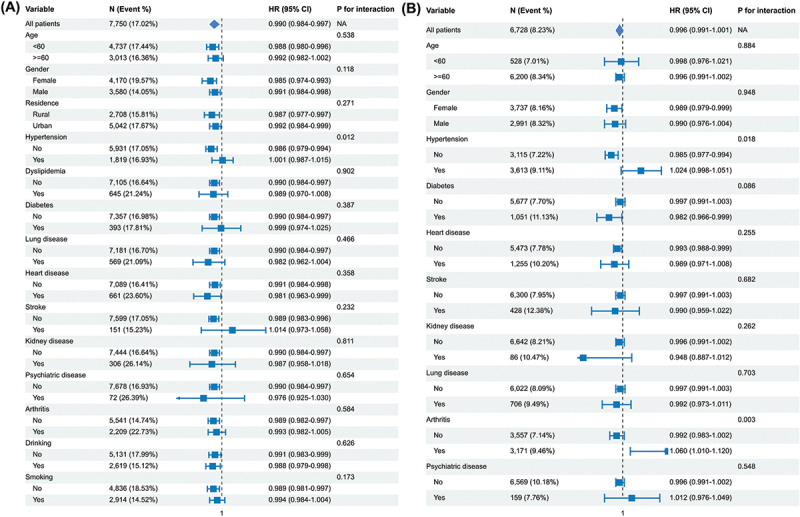
Subgroup analyses of the association between combined handgrip strength (CHS, per 1 kg increase) and incident outcomes, based on the fully adjusted model 2. (A) CHARLS cohort, incident chronic digestive system diseases. (B) SHARE cohort, incident peptic ulcer disease. Squares represent subgroup-specific hazard ratios; the diamond marker indicates the overall estimate; horizontal bars show 95% confidence intervals. *p*-values for interaction were derived from multiplicative interaction terms in the fully adjusted model.

Pre-specified subgroup analyses were conducted in both cohorts based on the fully adjusted Model 2 to explore potential effect modification (Figure 5). In the CHARLS cohort (Figure 5A), the inverse association between CHS and incident chronic digestive system diseases was directionally consistent across most subgroups. A statistically significant interaction was detected with hypertension status (*P*_interaction = 0.012). Stratified analyses showed that the inverse association was confined to participants without hypertension (HR, 0.986; 95% CI, 0.979–0.994), whereas no significant association was observed among those with hypertension (HR, 1.001; 95% CI, 0.987–1.015). No other pre-specified subgroup variable showed a statistically significant interaction with CHS in the CHARLS cohort.

In the SHARE cohort (Figure 5B), two statistically significant interactions were observed. Consistent with the CHARLS findings, an interaction with hypertension status was observed (*P*_interaction = 0.018), with the inverse association evident only among participants without hypertension (HR, 0.985; 95% CI, 0.977–0.994). An interaction with arthritis was also detected (*P*_interaction = 0.003): among participants without arthritis, CHS was not significantly associated with peptic ulcer risk (HR, 0.992; 95% CI, 0.983–1.002), whereas among participants with arthritis, higher CHS was associated with an increased risk (HR, 1.060; 95% CI, 1.010–1.120). This subgroup finding should be interpreted as exploratory and hypothesis-generating.

## Discussion

This parallel comparative study, based on two large, independent cohorts from China and Europe, provides prospective evidence that lower CHS is associated with incident self-reported digestive outcomes in middle-aged and older adults. The findings suggest a layered pattern: in the Chinese population (CHARLS), CHS was inversely associated with a broad, heterogeneous endpoint of ‘chronic digestive system diseases.’ In the older, clinically more complex European population (SHARE), the association was more specific and pointed to an inverse relationship between CHS and incident peptic ulcer disease, although statistical significance was mainly limited to female participants and short-term follow-up. This dual-layered evidence adds to current understanding of the link between musculoskeletal and digestive health while highlighting the complexity of this association across different disease spectra and population contexts.

The findings from the CHARLS cohort align with broader evidence linking low handgrip strength to various chronic conditions, including all-cause mortality, cardiovascular disease, and type 2 diabetes [11,14–18]. This suggests that reduced physiological reserve and systemic vulnerability reflected by lower muscle strength may also be relevant to digestive morbidity [19]. Given that the CHARLS outcome was a composite endpoint covering a range of functional and organic conditions, its association with CHS may reflect systemic pathophysiological processes related to sarcopenia and frailty. One possible mechanism is chronic low-grade inflammation, or ‘inflammaging’ [24,25]. As a critical endocrine organ, skeletal muscle dysfunction may be accompanied by reduced secretion of anti-inflammatory myokines and higher levels of pro-inflammatory cytokines. This imbalance may impair gastrointestinal barrier function and increase susceptibility to a variety of digestive diseases [26–29]. This interpretation is consistent with the emerging concept of the ‘gut–muscle axis,’ which describes a bidirectional regulatory relationship among the gut microbiota, systemic inflammation, and muscle metabolism [30–32].

In contrast, the association between CHS and peptic ulcer disease risk in SHARE requires more cautious interpretation, as the overall association was weak and appeared context-dependent. In the subgroup analysis, higher CHS was not clearly associated with PUD risk among participants without arthritis but was associated with a higher risk among those with arthritis. This finding should be considered exploratory and hypothesis-generating rather than confirmatory. One possible explanation is residual confounding by analgesic or NSAID use, as NSAIDs are commonly used for arthritis-related pain and are established contributors to peptic ulceration [5,33–35]. However, medication exposure was not available in the present analysis, and this pathway could not be directly tested. Therefore, the arthritis-related pattern should be interpreted as a signal requiring further investigation rather than as evidence that higher muscle strength increases PUD risk.

The hypertension-stratified pattern also warrants caution. In both cohorts, the inverse association between CHS and incident digestive outcomes was mainly observed among participants without hypertension; however, this should not be interpreted as evidence of a confirmed shared mechanism. This pattern may reflect differences in vascular, metabolic, inflammatory, or medication-related profiles between participants with and without hypertension. Although the renin–angiotensin system is biologically relevant to hypertension and has been implicated in intestinal inflammation, RAS activity, antihypertensive medication use, and related biomarkers were not directly measured in this study [36–38]. Accordingly, the hypertension-related findings should be regarded as exploratory and hypothesis-generating.

The main strengths of this study include its prospective design, large sample size, use of two independent ageing cohorts, and parallel assessment of broad and specific digestive outcomes. The use of CHS provided a bilateral measure of muscle strength, and sensitivity analyses using MSHS yielded directionally consistent results, particularly in CHARLS and in the overall pattern of SHARE, supporting the consistency of the main findings. Nevertheless, several limitations should be acknowledged. First, the outcome definitions differed between cohorts and were based on self-reported physician diagnoses. CHARLS captured a broad category of chronic digestive system diseases, whereas SHARE captured peptic ulcer disease only; therefore, the two analyses should be interpreted as complementary rather than directly comparable. Second, incident outcomes were recorded at discrete follow-up waves, and the exact date of disease onset was unavailable, although follow-up-window sensitivity analyses were performed to evaluate the stability of the findings. Third, the ROC-derived cut-off values were exploratory and cohort-specific; their discriminatory performance was modest, and conventional ROC analysis does not fully account for time-to-event outcomes. Fourth, key confounders, including nonsteroidal anti-inflammatory drug use, *Helicobacter pylori* infection, dietary intake, and objective nutritional biomarkers, were not consistently available, limiting mechanistic and causal interpretation. Finally, subgroup analyses should be considered exploratory because they may be affected by multiple comparisons, and the generalisability of the findings to younger populations remains uncertain.

From a clinical and public health perspective, handgrip strength remains attractive because it is simple, inexpensive, non-invasive, and feasible in community and primary-care settings. However, the present findings do not support using CHS as a stand-alone screening or diagnostic tool for digestive diseases. Rather, CHS may be considered a general marker of physiological vulnerability that could complement, but not replace, clinical assessment. The ROC-derived thresholds in this study should be viewed as exploratory reference values rather than clinically validated cut-offs. Future studies should validate these findings in cohorts with clinically confirmed outcomes, repeated grip strength measurements, detailed medication and nutritional data, and information on *Helicobacter pylori* infection before handgrip strength is considered for digestive disease risk assessment in practice.

## Conclusions

In this prospective parallel analysis of two large ageing cohorts, lower CHS was associated with subsequent self-reported digestive outcomes, with a more consistent inverse association for broad chronic digestive system diseases in CHARLS and a weaker, context-dependent association with peptic ulcer disease in SHARE. Sensitivity analyses using alternative follow-up windows and MSHS generally supported the direction of the main findings, although outcome heterogeneity, reliance on self-reported diagnoses, modest ROC performance, and unmeasured confounding limit causal and clinical interpretation. These results support further investigation of handgrip strength as a simple marker of physiological vulnerability related to digestive morbidity in older adults.

## Supplementary Material

Supplementary_File_clean.docx

## Data Availability

The data analysed in this study were derived from CHARLS and SHARE. Both datasets are publicly available for scientific use upon completion of a registration and application process at their respective websites: http://charls.pku.edu.cn/ for CHARLS and http://www.share-project.org/ for SHARE.

## Preprint statement

This manuscript has not been posted on any preprint server.
