# Supplementary material for: Combined handgrip strength and incident chronic digestive diseases: a prospective parallel analysis examining broad and specific endpoints in Chinese and European older adults
Source: Glob Health Action. 2026 Jun 15;19(1):2686499. doi: 10.1080/16549716.2026.2686499 (PMC13270866; doi:10.1080/16549716.2026.2686499)
Supplement: Supplementary_File_clean.docx [file ZGHA_A_2686499_SM3059.docx]

**Supplementary Tables**

**Table S1. Baseline characteristics of participants in the SHARE cohort (n = 6,728)**

| **Characteristic** | **Overall (n = 6,728)** | **Women (n = 3,737)** | **Men (n = 2,991)** | **P value** |
| --- | --- | --- | --- | --- |
| Age (years), mean ± SD | 65.9 ± 8.0 | 65.5 ± 8.4 | 66.4 ± 7.6 | <0.001 |
| Married, n (%) | 4,798 (71.3) | 2,447 (65.5) | 2,351 (78.6) | <0.001 |
| Urban residence, n (%) | 4,414 (65.6) | 2,469 (66.1) | 1,945 (65.0) | <0.001 |
| Education level (high), n (%) | 1,672 (24.8) | 850 (22.7) | 822 (27.5) | <0.001 |
| Smoking, n (%) | 1,098 (16.3) | 548 (14.7) | 550 (18.4) | <0.001 |
| Drinking, n (%) | 2,739 (40.7) | 1,145 (30.6) | 1,594 (53.3) | <0.001 |
| BMI (kg/m²), mean ± SD | 26.7 ± 4.4 | 26.3 ± 4.7 | 27.1 ± 4.0 | <0.001 |
| Hypertension, n (%) | 2,996 (44.5) | 1,657 (44.3) | 1,339 (44.8) | <0.001 |
| Diabetes, n (%) | 784 (11.7) | 379 (10.1) | 405 (13.5) | 0.041 |
| Stroke, n (%) | 267 (4.0) | 143 (3.8) | 124 (4.1) | <0.001 |
| Heart disease, n (%) | 866 (12.9) | 399 (10.7) | 467 (15.6) | 0.005 |
| Kidney disease, n (%) | 86 (1.3) | 46 (1.2) | 40 (1.3) | 0.028 |
| Lung disease, n (%) | 476 (7.1) | 260 (7.0) | 216 (7.2) | <0.001 |
| Psychiatric disease, n (%) | 159 (2.4) | 96 (2.6) | 63 (2.1) | 0.679 |
| Arthritis, n (%) | 2,209 (32.8) | 1,469 (39.3) | 740 (24.7) | <0.001 |

**Note.** Data are presented as mean ± standard deviation (SD) or number (n) with percentage (%). P values were calculated using the independent t-test for continuous variables and the chi-squared (χ²) test for categorical variables to compare differences between women and men.

**Table S2. Association of combined handgrip strength and maximal single-hand grip strength with incident peptic ulcer disease in the SHARE cohort**

| **Exposure** | **Population** | **Main analysis** |  |  | **Sensitivity analyses** |  |  |  |
| --- | --- | --- | --- | --- | --- | --- | --- | --- |
|  |  | **n/events** | **Model 1 HR (95% CI)** | **Model 2 HR (95% CI)** | **n/events** | **First follow-up wave HR (95% CI)** | **n/events** | **First two follow-up waves HR (95% CI)** |
| CHS, continuous | Overall | 6,728/554 | 0.994 (0.989-0.999) | 0.996 (0.991-1.001) | 6,728/218 | 0.994 (0.986-1.002) | 6,728/363 | 0.994 (0.988-1.000) |
|  | Men | 2,991/249 | 0.986 (0.973-1.000) | 0.990 (0.976-1.004) | 2,991/104 | 0.981 (0.963-1.004) | 2,991/151 | 0.989 (0.974-1.005) |
|  | Women | 3,737/306 | 0.988 (0.976-0.997) | 0.989 (0.979-0.999) | 3,737/114 | 0.982 (0.971-0.996) | 3,737/212 | 0.991 (0.980-1.003) |
| CHS group | Low | 2,766/307 | Reference | Reference | 2,766/137 | Reference | 2,766/227 | Reference |
|  | High | 3,962/247 | 0.751 (0.554-0.958) | 0.785 (0.614-1.005) | 3,962/81 | 0.668 (0.461-0.968) | 3,962/136 | 0.822 (0.606-1.115) |
| MSHS, continuous | Overall | 6,728/554 | 0.990 (0.982-0.999) | 0.993 (0.984-1.002) | 6,728/218 | 0.988 (0.975-1.001) | 6,728/363 | 0.991 (0.981-1.001) |

**Note.** Data are presented as hazard ratios (95% confidence intervals). Model 1 was the unadjusted crude model. Model 2 was the fully adjusted model including age, sex, marital status, residence, education level, smoking status, alcohol consumption, body mass index, hypertension, diabetes, heart disease, stroke, kidney disease, chronic lung disease, psychiatric disease, and arthritis. In sex-stratified analyses, sex was not included as an adjustment variable. Sensitivity analyses used the same covariates as Model 2 but restricted outcome ascertainment to the first follow-up wave or to the first two follow-up waves. CHS was defined as the sum of the maximum grip strength values from the left and right hands. MSHS was defined as the maximum grip strength value from either hand. The MSHS analysis is presented as a simplified reference sensitivity analysis using continuous exposure in the overall population. CHS, combined handgrip strength; MSHS, maximal single-hand grip strength; HR, hazard ratio; CI, confidence interval; SHARE, Survey of Health, Ageing and Retirement in Europe.

**Table S3. Sensitivity analysis using handgrip strength asymmetry in the CHARLS and SHARE cohorts**

| **Cohort** | **Outcome** | **Model** | **HGS asymmetry group** | **No. of events / participants** | **HR** | **95% CI** | **P value** |
| --- | --- | --- | --- | --- | --- | --- | --- |
| CHARLS | Incident chronic digestive system diseases | Model 1 | No (ratio ≤1.10) | 566 / 4,605 | Reference | — | — |
| CHARLS | Incident chronic digestive system diseases | Model 1 | Yes (ratio >1.10) | 435 / 3,145 | 1.14 | 1.01–1.29 | 0.038 |
| CHARLS | Incident chronic digestive system diseases | Model 2 | No (ratio ≤1.10) | 566 / 4,605 | Reference | — | — |
| CHARLS | Incident chronic digestive system diseases | Model 2 | Yes (ratio >1.10) | 435 / 3,145 | 1.08 | 0.95–1.23 | 0.242 |
| SHARE | Incident peptic ulcer disease | Model 1 | No (ratio ≤1.10) | 252 / 3,246 | Reference | — | — |
| SHARE | Incident peptic ulcer disease | Model 1 | Yes (ratio >1.10) | 302 / 3,482 | 1.12 | 0.95–1.32 | 0.182 |
| SHARE | Incident peptic ulcer disease | Model 2 | No (ratio ≤1.10) | 252 / 3,246 | Reference | — | — |
| SHARE | Incident peptic ulcer disease | Model 2 | Yes (ratio >1.10) | 302 / 3,482 | 1.07 | 0.90–1.27 | 0.456 |

**Note.** Data are presented as hazard ratios (HRs) with 95% confidence intervals (CIs) and P values from Cox proportional hazards models; the reference group in each model is participants without handgrip strength asymmetry (ratio ≤1.10). Model 1 was the unadjusted crude model. Model 2 used the same covariate set as Model 2 in the main analysis (Methods, Section 2.5): in CHARLS, age, sex, marital status, residence, smoking status, alcohol consumption, body mass index, hypertension, dyslipidemia, diabetes, heart disease, stroke, kidney disease, chronic lung disease, psychiatric disease, and arthritis; in SHARE, the same covariates with educational attainment added and dyslipidemia omitted (not captured in SHARE). Analytical samples, outcomes, follow-up, and censoring were identical to those used in the main analyses. Handgrip strength asymmetry. The asymmetry ratio was calculated as max(Left_max, Right_max) / min(Left_max, Right_max), and a ratio >1.10 was used to define asymmetry, in line with McGrath et al. and Collins et al. Because hand dominance was not recorded in CHARLS or SHARE, the max/min ratio was used as a hand-dominance-independent operationalization equivalent to inverting all dominant/non-dominant ratios to ≥1.0 prior to thresholding. Abbreviations. CHARLS, China Health and Retirement Longitudinal Study; SHARE, Survey of Health, Ageing and Retirement in Europe; HGS, handgrip strength; HR, hazard ratio; CI, confidence interval.
